# In situ imaging of intracellular miRNAs in tumour cells by branched hybridisation chain reaction

**DOI:** 10.1111/cpr.13721

**Published:** 2024-07-22

**Authors:** Ying Tang, Siwei Zhang, Xinyu Yang, Yao Chen, Sha Chen, Qiang Xi, Long Chao, Zhao Huang, Libo Nie

**Affiliations:** ^1^ Hunan Key Lab of Biomedical Materials and Devices, College of Life Sciences and Chemistry Hunan University of Technology Zhuzhou China; ^2^ State Key Laboratory of Chemo/Biosensing and Chemometrics, College of Chemistry and Chemical Engineering Hunan University Changsha China; ^3^ Zhuzhou City Joint Laboratory of Environmental Microbiology and Plant Resources Utilization Zhuzhou China; ^4^ Hunan Prevention and Treatment Institute for Occupational Diseases Changsha China

## Abstract

The ability to visualise microRNA in situ is crucial for studying microRNAs, their microRNA‐associated biological functions and disease diagnosis. Traditional fluorescence in situ hybridisation methods based on paraformaldehyde fixation of microRNAs suffer from release of microRNAs from cells, which limits the sensitivity of in situ hybridisation, making them unsuitable for the detection of small, low‐abundance microRNAs. To reduce the loss, microRNAs were covalently cross‐linked to proteins within cells by combining EDC and paraformaldehyde, and the target microRNA was used as the initiator chain for a branched hybridisation chain reaction to detect microRNA expression levels in situ. A simplified branched hybridisation chain reaction can be realised by coupling two hybridisation chain reaction circuits with a hairpin linker. Upon forming the primary hybridisation chain reaction product with extended sequence, this sequence reacts with the linker hairpin H3 to release the initiator sequence, resulting in the formation of numerous dendritic branched hybridisation chain reaction products. Imaging results show that this technique can detect microRNAs with high sensitivity and selectivity at both the single‐cell and single‐molecule levels. Compared with the traditional fluorescence in situ hybridisation technique, this method greatly improves the sensitivity and image resolution of in situ imaging detection. Therefore, we believe that the target‐initiated branched hybridisation chain reaction based in situ detection method provides a reliable assay platform for analysing disease‐related microRNA expression.

## Graphical abstract

The cross‐linked miRNA is used as the initiator chain for bHCR to visualisation of miRNA expression levels in situ.

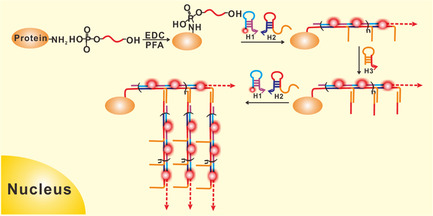

## 1 INTRODUCTION

MicroRNA (miRNA) is a small non‐coding RNA with a length of 19–25 nucleotides that play an important role in expression of post‐regulated transcriptional genes in organisms.^1^, ^2^ The dynamic changes of miRNA expression are related to the type and stage of the disease, and also the response to treatment and the state of stem cell differentiation.^3^, ^4^, ^5^, ^6^, ^7^, ^8^ It has been suggested in several studies that miRNA expression profiling can be a powerful tool for identifying clinical characteristics of tumours, including the source of the tumour, the differentiation process, the invasion process and the response to therapy.^9^, ^10^ As well as providing candidates for biomarkers used in cancer diagnose, prognosis, and treatment, it also screens for potential target molecules that can be treated with drugs and genes.^11^, ^12^, ^13^ Therefore, it is of great significance to develop sensing techniques for in situ mapping of specific miRNA expression levels in single cells. Currently, techniques based on Northern blotting, RT‐PCR, and microarray hybridisation have been extensively developed for analysis of miRNAs expression profiles.^14^, ^15^, ^16^, ^17^, ^18^ However, it is difficult for most of these single‐binding probe output detection methods to detect intracellular miRNAs, especially in single cell resolution due to their small size, low in abundance and highly homologous sequences.^19^ In addition, these methods are population‐based, which require the lysis of many cells to detect the average miRNA expression level, rather than evaluating miRNA abundance at the single‐cell level.^20^ Thus, it is essential to develop an in situ assay methods to detect the expression of miRNAs at the single‐cell level.

At present, fluorescence in situ hybridisation (FISH) is the most commonly used technique to study the in‐situ gene expression in cells.^21^ One disadvantage of this method is the lack of signal amplification, which limits the detection sensitivity of in situ hybridisation and makes it unsuitable for low‐abundance miRNA expression. As an in situ isothermal nucleic acid amplification technique, rolling circle amplification (RCA)‐based FISH is a method for detecting DNA, mRNA, and miRNA with high sensitivity. By using a single primer, DNA polymerase can generate hundreds of copies of the tandem sequence within minutes.^22^ RCA‐based FISH has, however, a limited detection efficiency due to the ligation of circular probes in situ and the significant hindrance of amplification reaction inside the cells by protein topology or binding proteins.^23^, ^24^ Moreover, the involvement of polymerase complicates the reactions and increases the cost. To address above issues, non‐enzymatic hybridisation chain reaction (HCR) has emerged as an excellent technique, in which amplification of signals does not require enzymes. This approach has been successfully applied to the quantitative detection of RNAs.^25^, ^26^, ^27^ Miao developed an electrochemical biosensor utilising a dumbbell HCR for the ultrasensitive detection of exosomal miRNA.^27^ This novel HCR, coupled with dual signal amplification, enhances the sensitivity of exosomal miRNA detection, and the method has been successfully applied to the analysis of cell and serum samples. In order to improve the efficiency of in situ imaging of individual mRNA mutations in single cells, our group has developed a new hybridisation technique for FISH called branched hybridisation chain reaction FISH (bHCR‐FISH), which eliminates the need for stringent hybridisation conditions for HCR polymerisation.^28^ Using this technique, it is possible to achieve greater sensitivity and amplification gain under more stringent hybridisation conditions than standard HCR when a highly branched product is formed, rather than a standard HCR.

**FIGURE 1:**
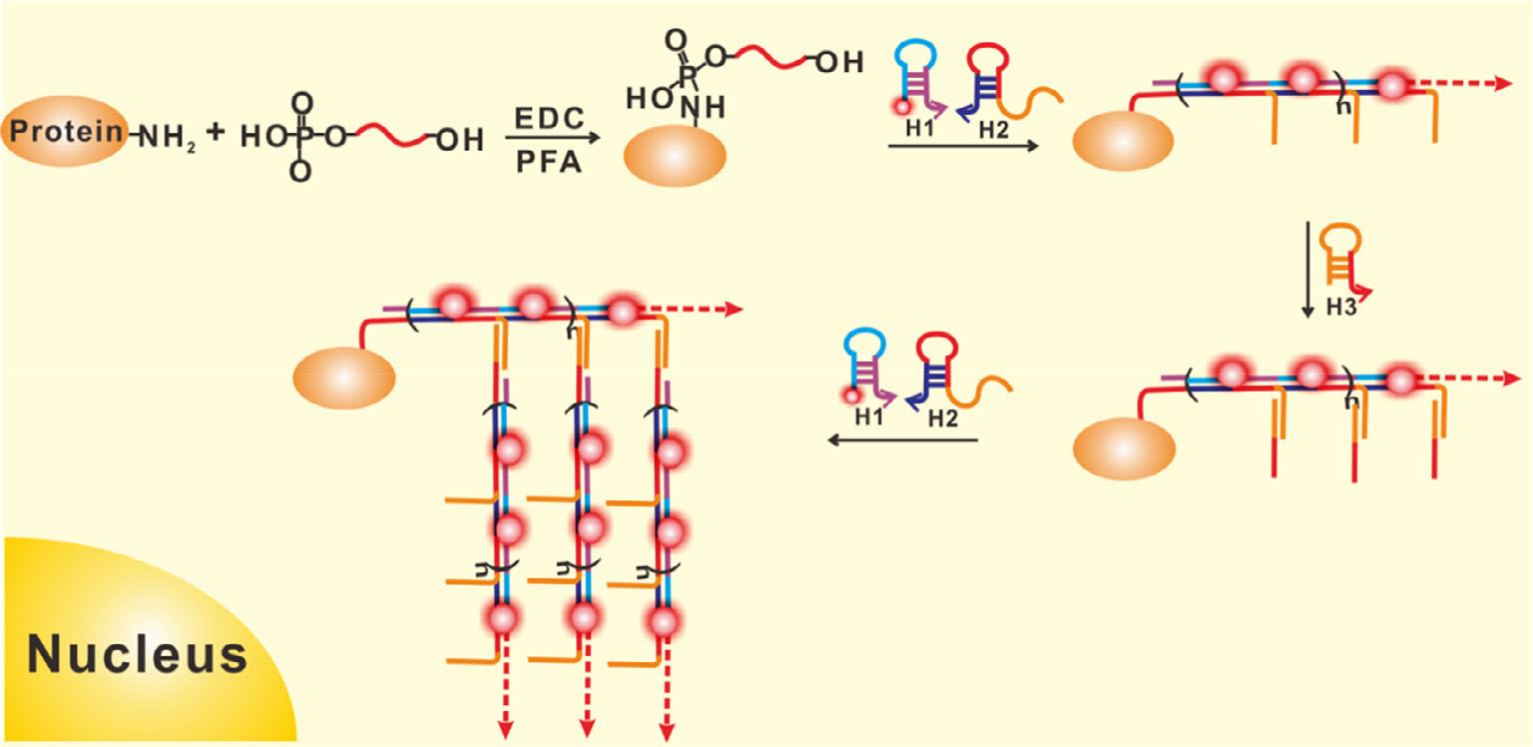
Schematic illustration of individual miR‐21 imaging in single cells using bHCR‐based FISH.

Herein, the bHCR has been used to quantify miRNA expression at the single cell level in cancer cells using in situ imaging. This FISH experiments, two key ideas are employed: one for covalently fixing miRNAs in cells, and the other for amplifying the signal of each target miRNA with bHCR, as illustrated in Figure 1. Due to the low abundance of miRNAs and their small size, traditional paraformaldehyde fixation of miRNAs suffers miRNA release from cells.^18^ In order to improve miRNA retention in cells, miRNAs were covalently cross‐linked to intracellular proteins by 1‐ethyl‐3‐(3‐dimethylaminopropyl) carbodiimide (EDC) after the formaldehyde treatment. This approach consists of three hairpin probes, a fluorophore labelled hairpin H1 and hairpin H2 with extension sequence and a linker hairpin H3 for subsequent bHCR detection. The fixed target miRNA is anchored on the protein matrix acting as the initiator triggers the hybridisation chain reaction to assemble the hairpins H1 and H2 to generate a primary polymeric polymer with extension sequences. The linker probe H3 is designed as a hairpin structure to block the initiator sequence, which can react with the side chain extension sequence along the HCR polymerisation products to expose the initiator. Meanwhile the newly exposed initiator would then trigger secondary HCR reaction with hairpin H1 and H2 to produce a highly branched fluorescent polymer, thus achieving the highly sensitive visual detection of the target miRNA. As a demonstration, we use this strategy assay for microRNA‐21 (miR‐21), which is overexpressed in cervical cancer, glioblastoma, cholangiocarcinoma, breast cancer, myeloma and other tumour cells.^29^ It was demonstrated that this method allows visualisation of miRNA at the single cell level and differentiation of miRNA expression levels within different cells to be highly sensitive and selective.

## 2 MATERIALS AND METHODS

### 2.1 Materials

RNase A, RPMI 1640 medium, foetal bovine serum, penicillin and streptomycin were obtained from Thermo Fisher Scientific (Waltham, MA, U.S.A.). 4,6‐Diamidino‐2‐phenylindole (DAPI), paraformaldehyde, diethyl pyrocarbonate (DEPC), tween‐20, EDC, formamide, sodium chloride/sodium citrate (SSC) buffer (DEPC treated, 20×), 1× PBS buffer (137 mM NaCl, 2.7 mM KCl, 10 mM Na_2_HPO_4_, 1.8 mM KH_2_PO_4_, pH 7.4), 5 × TBE buffer (225 mM tris‐boric acid, 50 mM EDTA, pH 8.0) and the oligonucleotides used in this work were purchased from Shanghai Sangon Biological Coporation (Shanghai, China). Human embryonic kidney cells (HEK‐293) and cervical cancer cells (HeLa) were obtained from the cell bank of Central Laboratory at Xiangya Hospital (Changsha, China). Listed in Table 1 are the synthesised oligonucleotides' sequences. RNA degradation was prevented by using DEPC and autoclaving all solutions and ultrapure water.

**TABLE 1:** Sequences of synthesised DNA probes.^a^

Name	Sequences (5′‐3′)
miR‐21	UAG CUU AUC AGA CUG AUG UUG A
H1	TCA AC *ATC AGT CTG ATA AGC TA* ACA AC *TAG CTT ATC AGA CTG AT*‐TAMRA
H2	GTT GAT GCA TTA GGT GGC GCT ACG ACT AAT GCA *TAG CTT ATC AGA CTG AT* GTT GA *ATC AGT CTG ATA AGC TA* GTT GT
H3	TAG CTT ATC AGA CTG AT *GTT GAT GCA TTA G* TCG TAG CGC CAC *CTA ATG CAT CAA C*
H4	TTA ACC *CAC GCC GAA TCC TAG ACT* CAA AGT *AGT CTA GGA TTC GGC GTG*‐TAMRA
H5	GGT TAA ACC ATT GTC TGG AAG GAA TCA CAA TGG T *AGT CTA GGA TTC GGC GTG* GGT TAA *CAC GCC GAA TCC TAG ACT* ACT TTG
H6	AGT CTA GGA TTC GGC GTG *GGT TAA ACC ATT GT* GAT TCC TTC CAG *ACA ATG GTT TAA CC*
FISH probe	TCA ACA TCA GTC TGA TAA GCT A‐TAMRA

^a^ Sequences in italics indicate complementary regions of the probes to form hairpin structure. Oligonucleotides are given in 5'‐3'order.

### 2.2 Apparatus and characterisation

Agarose gel electrophoresis images were captured via a Tocan 240 gel imaging system (Tocan, China). Atomic force microscopy (AFM) imaging was performed in air in a tapping mode on a Multimode 8 Bruker Bioscope system (Bruker, USA). Confocal fluorescence images were acquired on Olympus LX81 inverted microscope (Olympus, Japan) using a 100× objective lens with oil dipping. A He‐Ne laser (559 nm) was used as excitation source, and a 575–675 nm bandpass filter was used for fluorescence detection. Stacks of 10–15 slices were made at 0.3 μm intervals from the cells to create Z‐stack images.

### 2.3 Preparation of bHCR products in vitro

The HCR reaction under routine solution‐phase hybridisation conditions was performed in a 25 μL reaction mixture containing 100 nM miR‐21, 1 μM probes H1 and H2 in 1× TAE buffer solution (40 mM Tris, 20 mM acetic acid, 12.5 mM MgCl_2_, 2 mM EDTA, pH 8.0) and reacted at 37°C for 4 h. HCR under stringent solution‐phase hybridisation conditions was performed in a 25 μL reaction mixture containing 100 nM miR‐21, 1 μM probes H1 and H2 in 1× hybridisation buffer (2× SSC, 20% formamide) and reacted at 37°C for 4 h. The bHCR products were obtained by mixing 1 μM H3 with above linear HCR products at 37°C for 1 h followed by adding 5 μM H1 and H2 in 1× hybridisation buffer at 37°C for 4 h.

### 2.4 The culture and fixation of cells

The HeLa and HEK‐293 cells were cultured at 37°C in humidified incubators with 5% CO_2_ in modified RPMI‐1640 medium containing 10% foetal bovine serum, 100 IU/mL penicillin and 100 mg/mL streptomycin. It was then decided to seed the cells into plastic petri dishes with glass bottoms when the cells had reached 80% confluence, and they would be grown over night. After washing the cells twice with 1× PBS buffer, the cells were fixed for 30 min at room temperature in 1× PBS buffer containing 4% paraformaldehyde (PFA). After being washed twice with cold PBS, the cells were then incubated with freshly prepared 1× PBS buffer containing 0.13 M 1‐methylimidazole and 0.3 M NaCl for 10 min at room temperature. After removing the PBS solution, the cells were incubated for 1 h at 25°C with 500 L of EDC fixed solution (0.16 M EDC, 0.13 M 1‐methylimidazole, 300 mM NaCl, pH 8.0) and then washed twice with PBS to remove excess EDC. The fixed petri dishes were then dehydrated with 70%, 85% and 100% (vol/vol) ethanol for 3 min, respectively, and dried at room temperature. The fixed petri dishes could be stored in a refrigerator at −20°C for 1 week.

### 2.5 The bHCR FISH assay for miR‐21 in situ

Following are the procedures involved in performing the bHCR FISH assay for miR‐21. First, the fixed cells were rehydrated with 1× PBS buffer containing 0.05% tween‐20, after removing the PBS solution along with 500 μL of HCR reaction mixture including 1 μM H1 and H2, 1× reaction buffer (2× SSC, 20% formamide) were added and incubated at 37°C for 4 h. The excess probe should be washed with 1 mL of washing buffer at room temperature according to Choi's method.^27^

The second step was to anchor the linker probe to the above HCR product, adding 500 μL of reaction mixture including 1 μM probe H3 and 1× reaction buffer to the above petri dishes for an hour at 37°C, then washing with 1 mL PBS‐T three times for 10 min each time to remove excess probes.

The third step was to carry out the secondary HCR reaction, adding 500 μL of reaction mixture including 5 μM H1 and H2, 1× reaction buffer to above dishes and incubated at 37°C for 4 h, following the cells were washed in accordance with the first step. Before imaging, cells were stained with 1 μg/mL DAPI for 10 min at room temperature and washed three times with PBS.

### 2.6 Control experiments for bHCR FISH assay

Cells were pretreated with RNase A as a control experiment for bHCR‐FISH, in which fixed cells were pretreated with RNase A (50 μg/mL) at 37°C for 2 h, and then bHCR‐FISH experiments were performed.

A conventional FISH experiment were also conducted as a control for bHCR‐FISH, in which fixed cells were directly reacted with 1 μM FISH probe for 1 h at 37°C under 2× SSC and 20% formamide. Subsequently cells were washed as in step 1 in the bHCR‐FISH assay.

### 2.7 RT‐qPCR analysis of miR‐21 extracts from cells

RT‐qPCR analysis of miR‐21 extracts from Cells. Total RNA (40 μL) were extracted from HeLa and HEK‐293 cells using UNIQ‐10 column Trizol Total RNA Purification Kit (Bioengineering, Shanghai, China). The cDNA samples were prepared using the AMV First Strand cDNA Synthesis Kit (BBI, Toronto, Canada) as follows: 11 μL of reaction mixture containing 5 μL of total RNA, 1 μL of reverse transcription primer, and 5 μL of de‐nuclease water was incubated at 70°C for 5 min, followed by an ice bath for 10 sec. Then 1 μL of RNase inhibitor (20 U/μL), 2 μL of dNTPs (10 mM), 4 μL of 5× AMV reverse transcription buffer solution, 2 μL of AMV reverse transcriptase (10 U/μL) were added to the above reaction mixture, and the reaction was carried out at 37°C for 5 min, at 42°C for 1 h, and then inactivated at 70°C for 10 min.

RT‐qPCR analysis of miR‐21 was performed using SybrGreen PCR Master Mix (ABI, CA, USA) on an ABI StepOne plus qPCR (CA, USA) instrument according to the instructions. Briefly, a total volume of 20 μL reaction mixture containing 2 μL of 10‐fold diluted cDNA, 10 μL of SybrGreen PCR Master Mix, 0.4 μL of upstream primer (10 μM), 0.4 μL of downstream primer (10 μM), 7.2 μL of denuclease water. miR‐21 primer sequences were as follows: primer F: 5′‐ACA CTC CAG CTG GGT AGC TTA TCA GAC TG‐3′, and primer R: 5′‐TGGTGTCGTGGAGTCG‐3′. The qPCR reaction conditions were: denaturation at 95°C for 3 min, 95°C for 15 s, 40 cycles at 57°C for 20 s, and 72°C for 30 s. RT‐qPCR was performed in an ABI Stepone plus model fluorescence quantitative PCR instrument.

## 3 RESULTS

### 3.1 Characterisation of the bHCR products

**FIGURE 2:**
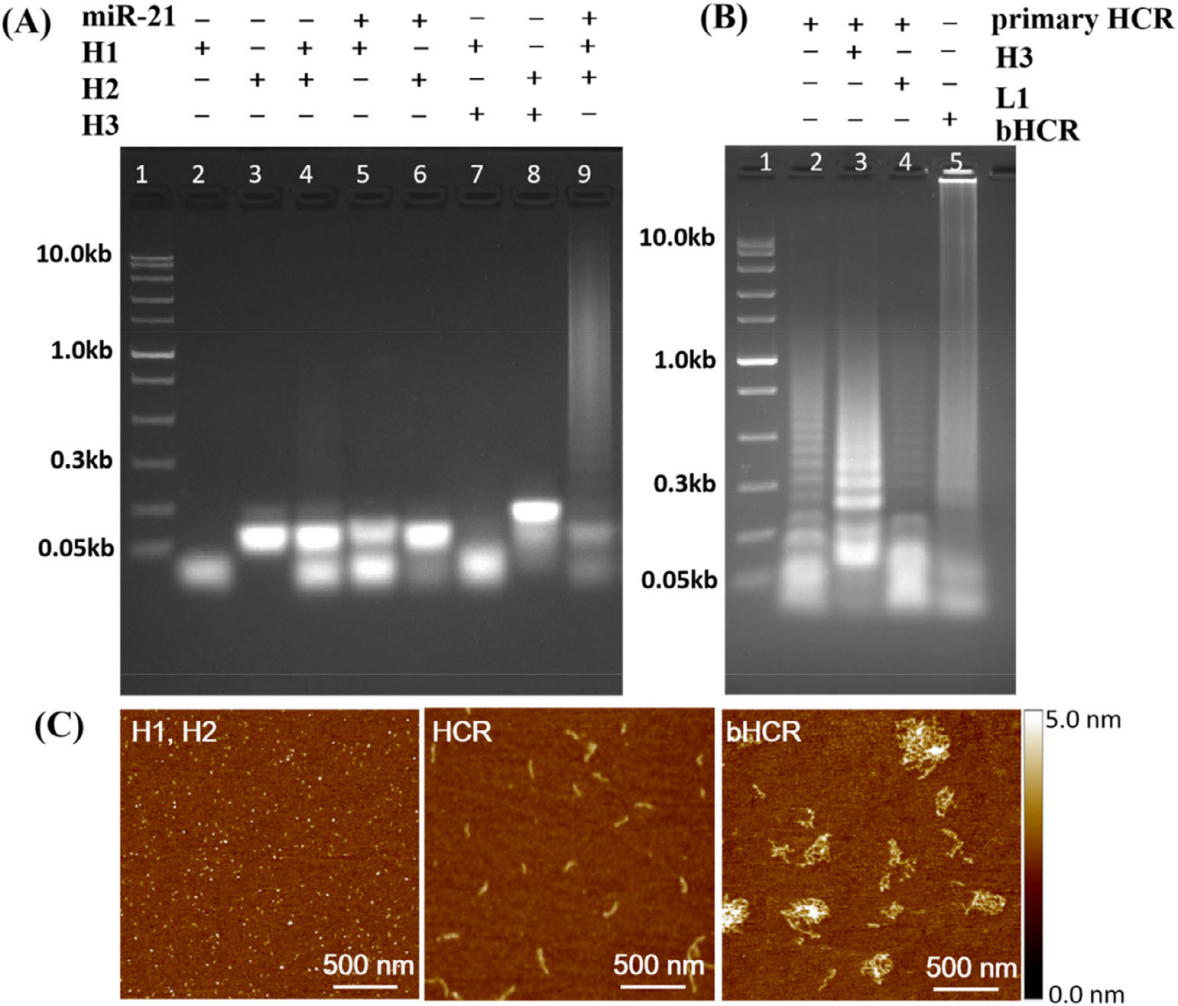
Amplification changes between HCR and bHCR. HCR amplification on gel electrophoresis under routine (A) and stringent hybridisation conditions (B). AFM images for HCR under stringent FISH conditions (C).

A gel electrophoresis was used to verify the feasibility of this strategy by comparing HCR under routine hybridisation conditions with bHCR under stringent hybridisation conditions. During routine hybridisation, gel electrophoresis results showed that a 1:10 ratio of target to hairpin probe hybridisation produced a broad molecular weight band ranging from ~300 bp to ~6 kbp (Figure 2A, Lane 9). The results showed that the gain efficiency of HCR amplification under routine hybridisation conditions was from ~4 to ~80. In contrast, HCR amplification under stringent hybridisation conditions, the only HCR products that were obtained were those with molecular weights below 1 kb (Figure 2B, Lane 2). HCR sensitivity and amplification efficiency were reduced by stringent hybridisation conditions, an observation consistent with previous studies.^30^ Branched HCR amplification was accomplished by adding the linker hairpin probe H3 along with the H1 and H2 hairpin probes, a bright band of the products with molecular weights over 10 kbp were observed (Figure 2B, Lane 5). The results directly prove that the gain efficiency of secondary HCR (bHCR) amplification under stringent hybridisation conditions was greater than 200 times. In FISH assays, bHCR showed high efficiency at amplification of signals. Initially, we designed a linearly linker probe L1 to trigger the secondary HCR amplification. As a result of the reaction between the primary HCR products and the linearly linked probe L1 resulted in the decomposition of most of the primary polymeric products into small polymers (Figure 2B, Lane 4). There may be a displacement of strands between the HCR polymer and the linearly linked probe L1, both of which have the same initiating sequence. Conversely, HCR polymer does not decompose in the hairpin probe H3. (Figure 2B, Lane 3). The reason may be that the initiation sequence was blocked by the hairpin, and the rigid double‐stranded structure prevents the intramolecular strand displacement when the promoter is exposed. These results indicate that bHCR can efficiently amplification under stringent hybridisation conditions, which enhancing in situ detection's sensitivity.

The formation of branched HCR products was further verified using atomic force microscopy (AFM), as shown in Figure 2C. Hairpin probes H1 and H2 maintained their stability under stringent hybridisation conditions without miR‐21 and appeared as tiny spots. When the target miR‐21 coexisted with hairpin probes H1 and H2, short linear HCR polymeric products with lengths of ~100 nm were obtained. After the secondary HCR amplification on the basis of the primary product, large linear agglomerate‐like polymeric products were obtained. In spite of the stringent FISH conditions, these images clearly demonstrated efficient branching of bHCR.

### 3.2 Investigation of miR‐21 fixation method

**FIGURE 3:**
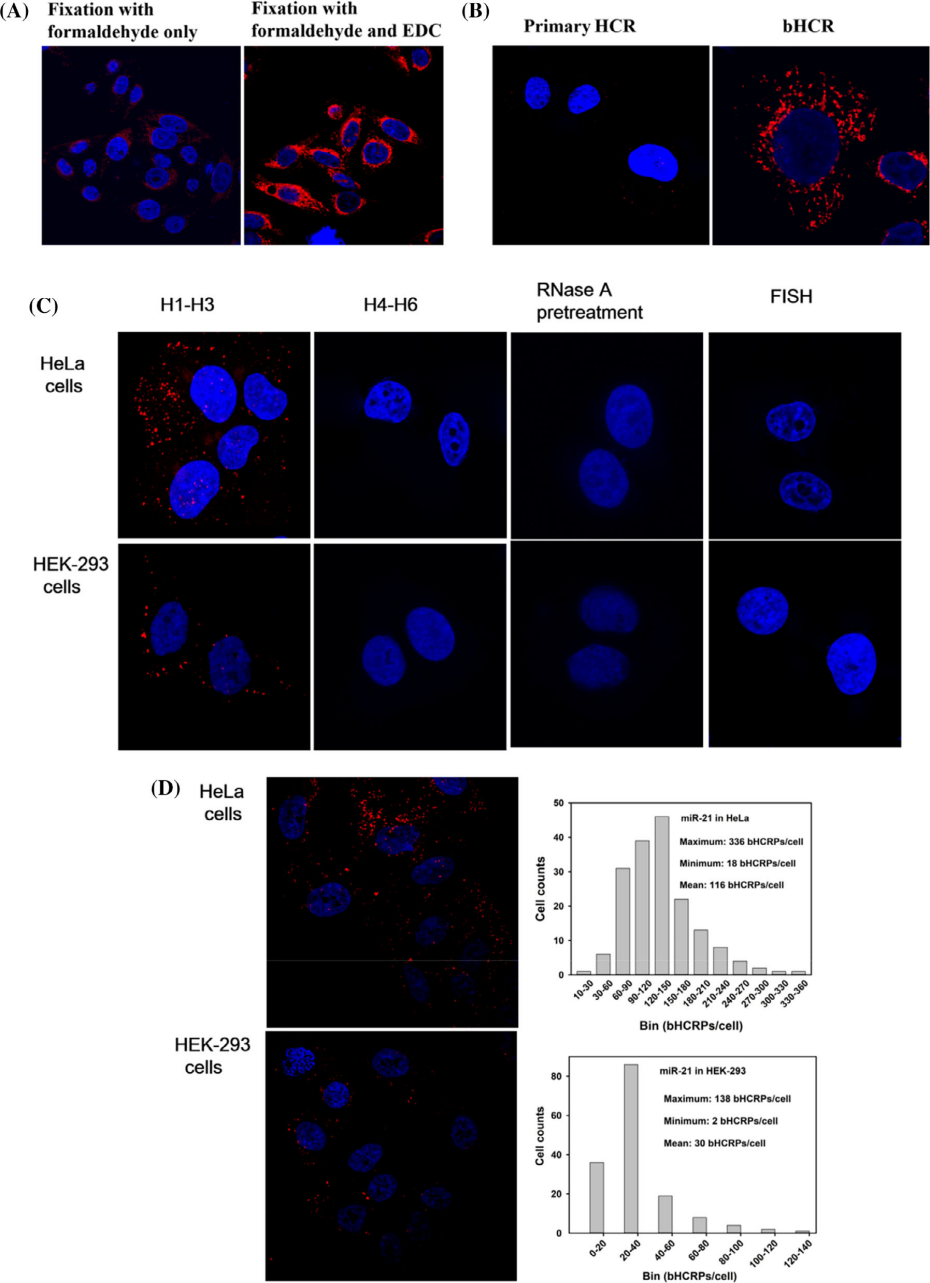
Fluorescence imaging of miR‐21 in different cells. (A) Investigation of miR‐21 fixation method. (B) HeLa cells after primary HCR amplification and secondary amplification. (C) bHCR‐based FISH detection of miR‐21 in HeLa and HEK‐293 cells. (D) Quantification of the average number of bHCRPs in HeLa and HEK‐293 cells.

Different fixation methods were investigated for miRNA detection. As shown in Figure 3A, Hela cells were selected as our target cells, according to the images, the samples fixed by traditional formaldehyde after bHCR reaction with multiple washing steps showed only weak fluorescence signals. However, many bright fluorescence signals were observed in cells modified by EDC crosslinking after formaldehyde fixation. These results suggest that the fixed cells lost most of their miRNAs during subsequent multi‐step experimental treatment. After cell paraformaldehyde fixation, additional EDC treatment was added for cross‐linking, in order to carry out irreversible cross‐linking between the phosphoric acid of RNA and the amino group of protein, so that the RNA was firmly fixed on the surrounding protein, which greatly reduced the loss during the experimental operation.

### 3.3 Sensitivity enhancement for bHCR

As mentioned above, because the amplification efficiency and sensitivity of standard HCR will be reduced under stringent in situ hybridisation conditions. Branched HCR was developed to produce a large number of fluorophores labelled polymeric products, which expected to obtain an enhancement sensitivity for miRNA fluorescence imaging in situ. To evaluate the method's enhanced sensitivity of detection, miR‐21 in HeLa cells were used as the target for in situ bHCR analysis. Based on the overlay images of different z‐axis scanning, only a few and dim fluorescent signal spots were observed in cells after primary HCR amplification (Figure 3B). On the contrary, when the cells followed secondary HCR amplification, the fluorescent spots in the cells were significantly enhanced, and the number was also increased compared with that of primary HCR amplification (Figure 3B). The results showed that bHCR avoid the loss of detection signals, and thus provide direct evidence for the enhanced sensitivity of bHCR than standard HCR.

### 3.4 Tumour cell miR‐21 fluorescence visualisation in situ

Next, bHCR method was used to investigate the expression level of miR‐21 in different cell lines. It is known that the expression level of miR‐21 in HeLa cells is relatively high whereas human embryonic kidney cells HEK‐293 is relatively low. In order to verify that bHCR can achieve in situ detection of miR‐21 in all cells with different expression levels, in situ fluorescence imaging analysis of bHCR for miR‐21 in HeLa and HEK‐293 cells were performed (Figure 3C). For HeLa cells treated with bHCR probes H1 to H3, many bright fluorescent spots were observed in the cytoplasm. In contrast, HEK‐293 cells with low miR‐21 expression showed significantly fewer fluorescence bright spots compared to HeLa cells. Using control probes H4 to H6, the cells were treated with bHCR, and none of the cells showed fluorescent spots in their cytoplasm. In order to further prove that the bHCR polymerisation products were induced by the target miR‐21, RNase A was used to pretreat the fixed cells, there was still no fluorescent bright spot in the cells. These results indicated that the fluorescent bright spots were specifically initiated by the target miRNA, thus achieving in situ fluorescence imaging of the target miRNA. Furthermore, this strategy were also compared with the conventional FISH method, because the conventional FISH did not amplify the signal, no corresponding fluorescence signal was observed in the cytoplasm. It was proved that efficient signal amplification by bHCR under in situ hybridisation was necessary to detect miRNA. Similar results were obtained for the detection of HEK‐293 cells with relatively low miR‐21 expression level. These results confirmed that the target miRNA‐induced bHCR method has high specificity and sensitivity.

Fluorescence images showed that single bright fluorescent spots from bHCR products (bHCRPs) were mostly randomly distributed in the cytoplasm, and no obvious aggregation was observed, which may correspond to individual miRNAs (Figure 3D). The number of bHCRPs varies greatly between cells, with the number of bHCRPs per cell in HeLa cells ranging from 18 to 336, with an average of 116. The number of bHCRPs per cell in HEK‐293 cells ranged from 2 to 138, with an average of 30. This variability suggests that miRNA expression may be different even within the same type of cell, as individual cells may be at different stages. The expression of miRNA‐21 in HeLa cells was significantly higher than that in HEK‐293 cells, which was consistent with the results of RT‐qPCR. As shown in Table 2, the expression level of miR‐21 in HEK‐293 cells was 0.366 times that of HeLa cells, and the data showed that the expression level of miR‐21 in HeLa cells was up‐regulated compared with HEK‐293 cells.

**TABLE 2:** Average *C*_*t*_ values in qPCR assay of miRNA‐21.

Cells	miR‐21	U6	∆*C*_*t*_	∆∆*C*_*t*_	2^−(∆∆Ct)^
HeLa	22.051	16.719	5.331	0	1
HEK‐293	23.712	16.933	6.778	1.447	0.366

**FIGURE 4:**
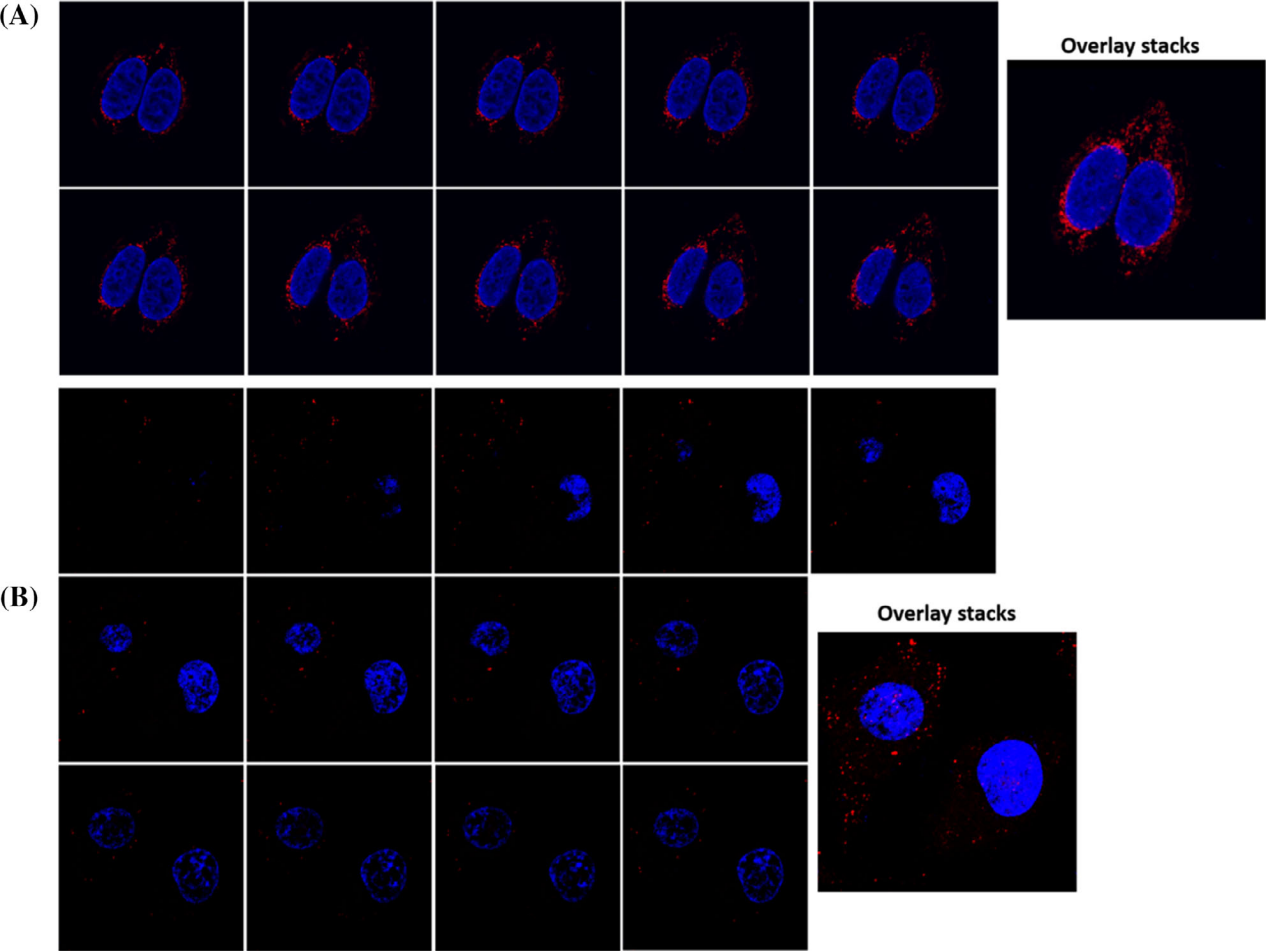
Z‐stacks analysis of target miRNA‐21 in HeLa cells (A) and HEK‐293 cells (B).

Since confocal imaging only detects fluorescent signals in the focal plane, z‐axis scanning was performed for the distribution of miR‐21 in cells. Branched HCR was applied to HeLa and HEK‐293 cells for the localisation of miR‐21 at different z‐axis cross sections. The results showed that the intensity and localisation of the fluorescence single spots in HeLa cells showed significant variations in different z‐axis cross sections (Figure 4A). The bright spots at the same position were the same miRNA molecules, and the intensity changes were from the sensitivity variations of HCR polymer detection at different z‐axis cross sections, while the bright spots at different positions represent different miRNA molecules. Similarly, z‐axis scanning of HEK‐293 cells also revealed the distribution of miRNA in the cells (Figure 4B). By comparing the overlay images of the two types of cells, it was found that miR‐21 expression in HeLa cells was higher than that in HEK‐293 cells.

## 4 DISCUSSION

miR‐21 is a member of the microRNA family, plays a crucial role in various cellular processes. Expression levels of miR‐21 have been found to be altered in various types of cancer, including cervical cancer. FISH is the most common used technique to study the in‐situ gene expression in cells.^21^ However, non‐specific adsorption poses a challenge in FISH experiments, as it can result in decreased sensitivity and interpretability of the results. One way to overcome this problem is through the use of multi‐step elution procedures. These procedures involve carefully removing non‐specifically bound molecules from the target of interest, often under strict conditions. However, these elution steps can lead to the degradation or loss of microRNAs. To overcome this challenge, certain signal amplification techniques are often employed during detection to enhance the sensitivity of the assay.

Crosslinking miRNA to nearby proteins using EDC effectively reduces the loss of miRNAs, enhancing their stability and allowing for accurate analysis. This technique has a wide range of applications and is a valuable tool in studying miRNA biology and function.^31^ By incorporating the fixed target miRNA as the initiator sequence, this method were able to initiate the bHCR reaction in a specific and localised manner. By using this signal amplification technique, the analysis of the expression of miR‐21 in HeLa cells and HEK‐293 cells revealed a significant difference in the level of expression between the two cell lines. The higher expression of miR‐21 in HeLa cells suggests that this microRNA may have different roles in cervical cancer cells compared to HEK‐293 cells. miR‐21 has been shown to regulate gene expression and promote cell growth, proliferation, and invasion in various types of cancer. The altered expression of miR‐21 in HeLa cells may indicate its role in the development and progression of cervical cancer.

## 5 CONCLUSIONS

In conclusion, this bHCR strategy for in situ detection of miRNA offers several advantages like enhanced sensitivity and imaging solution. By covalently cross‐linking target miRNA to intracellular proteins, it significantly restricts miRNA diffusion, thereby improving sensitivity. Furthermore, these immobilised target miRNA molecules initiate the bHCR reaction, leading to efficient signal amplification. This method not only demonstrates improved sensitivity compared to traditional FISH techniques but also offers enhanced imaging resolution. Successful application in analysing miR‐21 expression in HeLa and HEK293 cells underscores its efficacy. However, further research is needed to apply this method to tumour tissues, which will be a priority in our future work.

## AUTHOR CONTRIBUTIONS

**Ying Tang:** Conceptualisation; investigation; funding acquisition; writing—original draft. **Siwei Zhang:** Investigation; data curation; writing. **Xinyu Yang:** Validation; investigation; data curation. **Yao Chen:** Writing; validation; supervision; project administration; funding acquisition. **Sha Chen:** Supervision; writing—review & editing. **Qiang Xi:** Investigation; data curation. **Long Chao:** Resources; supervision. **Zhao Huang**: Resources; supervision; writing—review & editing. **Libo Nie:** Resources; supervision; writing—review & editing.

## CONFLICT OF INTEREST STATEMENT

The authors declare no competing financial interest.

## Data Availability

All data generated or analysed during this study are included in this published article and are available from the corresponding author upon reasonable request.
